# Foundation plant species provide resilience and microclimatic heterogeneity in drylands

**DOI:** 10.1038/s41598-022-22579-1

**Published:** 2022-10-26

**Authors:** C. J. Lortie, Alessandro Filazzola, Mike Westphal, H. Scott Butterfield

**Affiliations:** 1 Department of Biology, York University, Toronto, ON M3J1P3 Canada; 2 National Center for Ecological Analysis and Synthesis, University of California Santa Barbara, Santa Barbara, USA; 3 Centre for Urban Environments, University of Toronto Mississauga, Mississauga, ON L5L 1C6 Canada; 4 Bureau of Land Management, Central Coast Field Office, 940 2nd Avenue, Marina, CA 93933 USA; 5 The Nature Conservancy, 830 S Street, Sacramento, CA 95811 USA

**Keywords:** Climate-change ecology, Climate-change mitigation

## Abstract

Climate change profoundly influences plants and animals in all ecosystems including drylands such as semi-arid and arid scrublands and grasslands. At the peak of an extended megadrought in the Southwestern USA, the microclimatic refuges provided by foundation plant species and through associated vegetation were examined. Shrubs and open interstitial spaces without a canopy but with annual plants were instrumented in 2016 and the wet season of 2017 in the central drylands of California. In both years and all seasons tested, vegetation significantly mediated fine-scale near-surface air temperature and relative soil moisture content—defined here as microclimate. The foundation species with other vegetation provided the most significant thermal refuge potential capacity for other plants and animals, but there was variation by growing season. Soil moisture content was frequently increased by the direct canopy effects of shrubs. This evidence suggests that the climate many plants and animals experience, even during an extended megadrought, is mediated by the local plants in highly impacted drylands with anthropogenic disturbance and significant water-induced challenges. Foundation species such as shrubs in drylands function as a potent starting point in examining the ecological relevance of climate at scales germane to many species locally. An ecological framework for climate resilience using shrubs will improve conservation and restoration planning in drylands.

## Introduction

Climate change is palpable. In drylands including deserts and semi-arid grasslands and scrublands, the direct and indirect impacts of climate are profound^1^. Climate drivers relevant to plant, animals, and most dimensions of human interactions in these ecosystems are influenced through at least three key changes^2^. Increased mean temperatures that drive vegetation loss in drylands^1^. Increased variability in temperatures and precipitation^3–5^. Extended drought or megadroughts^6,7^ that impose environmental limitations and constraints on ecological resilience and function through water limitations for natural vegetation and for agriculture regionally^8^. In concert with this forcing from climate, vegetation with land use changes^9^ and groundwater processes^10–12^ strongly suggest that resilience is in jeopardy in terms of many ecosystem functions including biodiversity preservation^13^.

There are many solutions and mitigation strategies ranging from local to global^14^ and from policy and science^15^ to stakeholder restoration and management^16^. We propose that a simple solution at fine-scales is to design strategies that capitalize on existing and extant vegetation—particularly resident native species—and those fundamental to function such as foundation species. A foundation species is one that is not necessarily common but that provides critical support for ecological processes that structure community assembly and diversity patterns^17,18^. Here, surface air temperature and available soil moisture were examined, but more complex measures including water loss via the transpiration of plants can also be important ecological processes relevant to understanding the direct and indirect effects of foundation plant species in drylands. The hypothesis that plants can buffer climate change has been tested primarily through landscape-level analyses such as NDVI/vegetation land cover^19^ or in northern ecosystems^20,21^. However, foundation plant species such as shrubs that comprise a significant component of the structure in some of these drylands systems provide many key ecological functions and can also mitigate climate change effects in at least four micro-environmental capacities^22^.

The following contrasting predictions can be explored to examine this hypothesis through fine-scale structured ecological monitoring. (1) Shrubs decouple key micro-environmental measures of climate such as near-surface air temperature and soil moisture from open, non-canopied or interstitial microsites within a dryland region (i.e. distinct refuges)^23^. (2) Shrubs buffer the microclimate but changes between shrub-open contrasts move in tandem (reduced magnitude in stressors but similar trends seasonally)^24^. (3) Shrubs indirectly influence microclimate through facilitation of ground-covering plants (indirect vegetation buffering)^22^. (4) Finally, shrubs interact with microclimate in less linear or predictable capacities or do not necessarily provide a fine-scale refuge with positive influences on microclimate consistent with assumed and other reported ecological benefits^25^. Excepting the final null prediction, the overarching hypothesis posits that shrubs and vegetation provide critical heterogeneity in key climate drivers at fine-scales. Specifically, the direct effects of a canopy provide ecologically meaningful heterogeneity in conditions for both plants and animals in the soils, soil moisture profiles, and unique temperature regimes from other desert microsites. There are also a host of indirect interactions that can function to provide resilience through the differences in how other plants and the animals respond to these microenvironmental and simple structural effects in the functioning of natural community dynamics.

**Figure 1:**
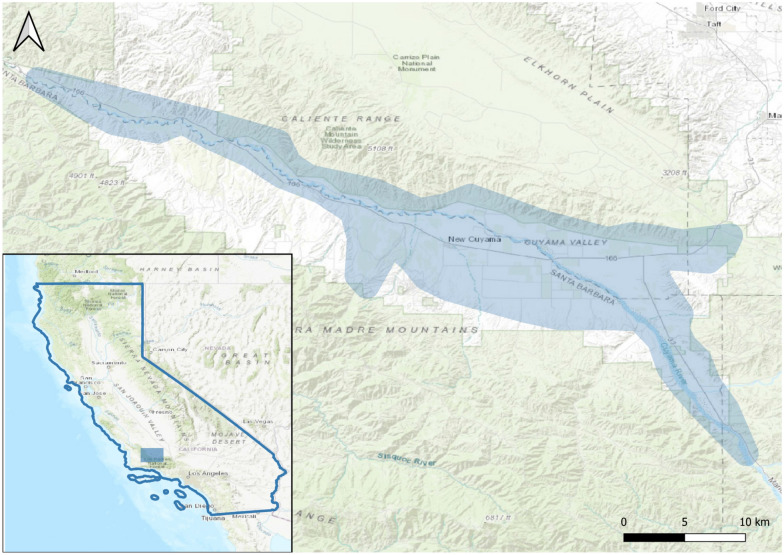
A map of Cuyama Valley in Central California drylands. This region is situated within the San Joaquin Desert of California between two inland mountain ranges. It is a major hydrological catchment with agriculture, energy developments, and extant native vegetation and animal species. All instances of remnant native *Ephedra californica* parklands with shrubs present and intact were sampled. Sets of shrubs and open, interstitial microsites were instrumented for assessment of microclimate changes regionally. All sites were located on the valley floor and were adjacent to anthropogenic disturbances such as agriculture, energy developments, or farms—but were mostly native patches of relict vegetation. The map of Cuyama Valley was rendered using Quantum GIS open-source software (www.qgis.org) version 3.12.1. The shaded relief map was from ESRI Topographical Maps (www.ESRI.com).

The relative importance of microclimatic refuges and heterogeneity was examined using foundation plant species, shrubs, in Cuyama Valley in the central drylands of California at the peak of the megadrought in the Southwestern North America (SWNA). This megadrought occurred from 2000 to 2018^7^. A megadrought is a particularly sustained and severe drought that spans many years and typically eclipses frequently observed dry episodes during 20th-century instrumental observations^6^. Cuyama Valley is centrally located within the interior drylands of California (Fig. 1). It encompasses arid and semi-arid habitats, and its ecological and anthropogenic value is significant regionally^26^. It is also challenged with varied hydrological limitations and water use environmental uncertainties^27^. It is thus a critical hotspot for microclimatic importance studies that examine the relative importance of salient and sensible assets ecologically such as vegetation. *Ephedra californica* is the dominant and most common shrub and native woody species within this region^28^, and it has low specific leaf areas^29^, long-lived^30^, low water needs for recruitment^31^, excellent recovery following disturbance^32^, and functions as a foundation species ecologically^33,34^. Accurate near-surface air temperatures with soil moisture content, structured to inform ecological processes, were used to inform a deeper understanding of microclimatic interactions that are germane to resident species^35^. Sensors were deployed under the shrubs and in the open, interstitial spaces without a canopy but with annual plants in all 12 months of 2016 and the 4 months of winter (or historical wet season in these drylands) for 2017. In half of the microsites, all aboveground non-woody vegetation was routinely clipped to further examine the importance of low lying, ground cover vegetation such as herbs, forbs, and grasses. This generated the following four microclimatic opportunities to instrument: shrub canopy, shrub with ground-covering vegetation, open microsites without a canopy or vegetation, and open microsite with ground-covering vegetation.

**Figure 2:**
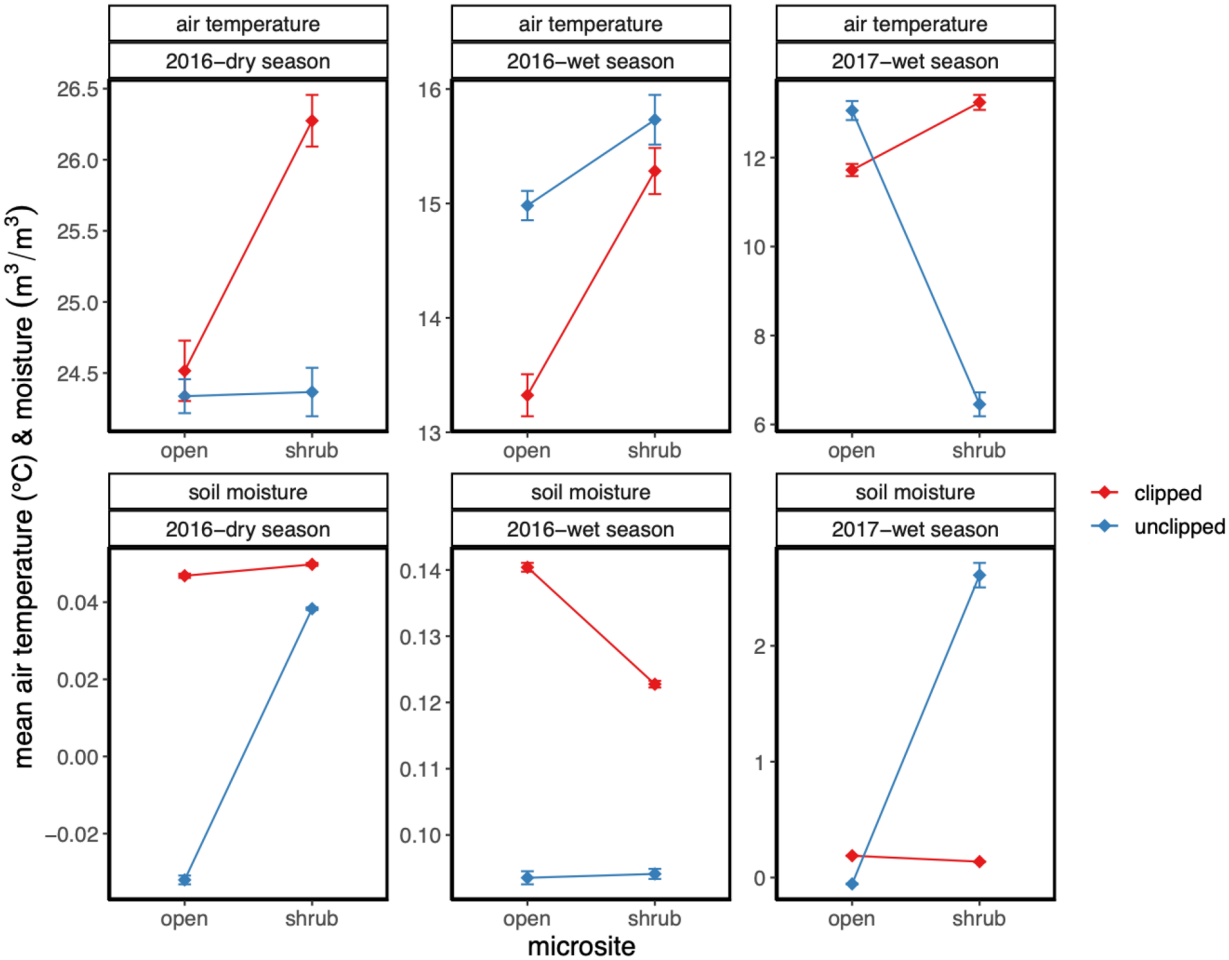
A contrast of the mean near-surface air temperatures and soil moisture content in Cuyama Valley, California in 2016 and the winter of 2017. Shrubs microsites are under the canopy of the resident native species *Ephedra californica*, and open are microsites without a woody canopy of a shrub. Clipped refers to the treatment of removing all aboveground non-woody vegetation both under shrubs and in the open. This sampling estimated microclimate at the peak of the SWNA megadrought. These plots depict seasonal means for each factor level (see Methods for delineations). Data models, statistical analyses, and visualization for this figure were done in R version 4.2.1 (see Supplement for full script and citation).

**Figure 3:**
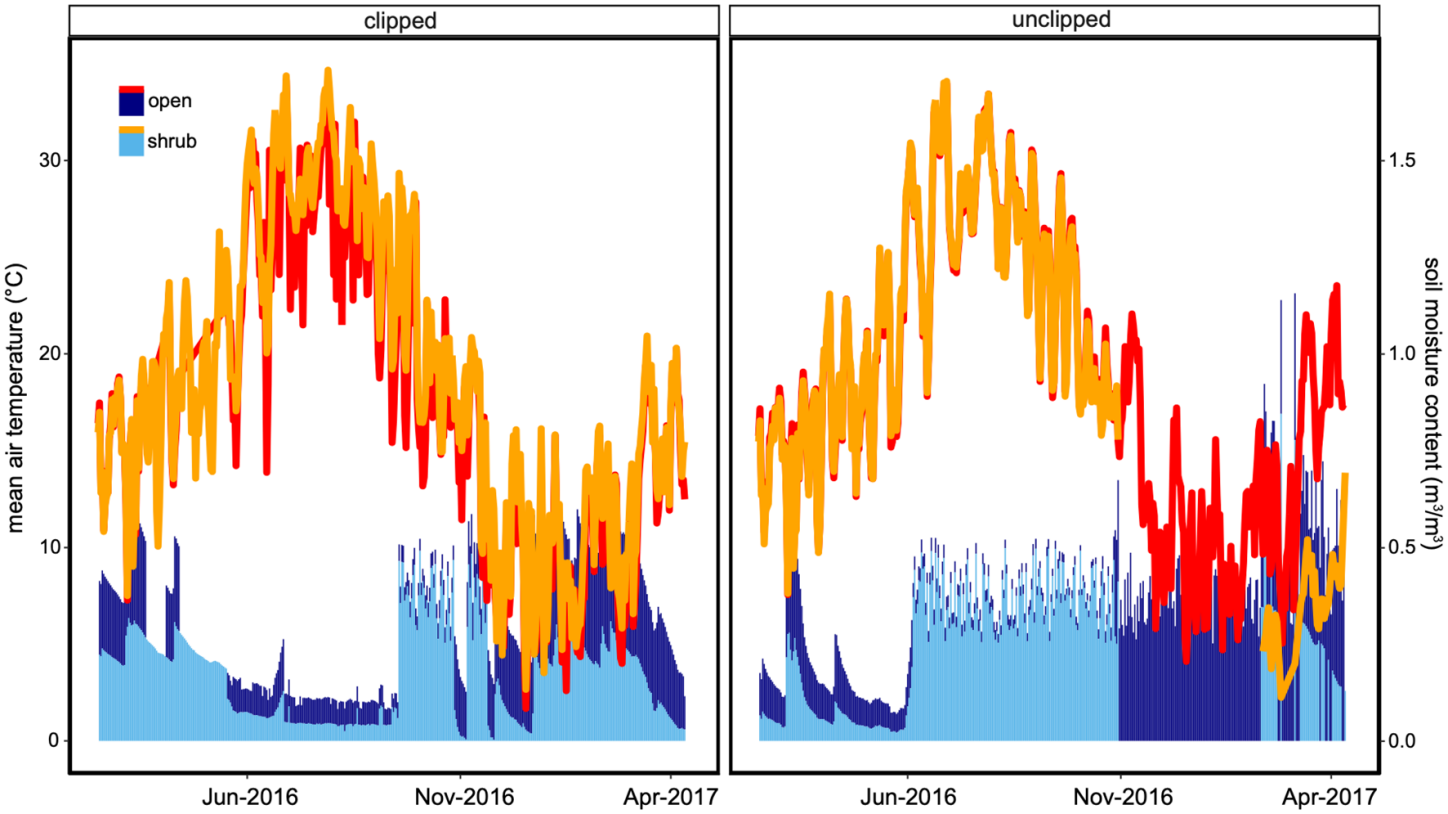
A climatograph of ecologically structured fine-scale climate observations instrumented in Cuyama Valley, California in 2016 and the winter of 2017. Near-surface mean air temperature and soil moisture were sampled under shrub canopy microsite and in the open without a canopy but with ground cover vegetation present. The clipped versus unclipped panels describe a treatment removing all non-woody vegetation from near instrumentation in each microclimatic content examined. These data were collected during the extended SWNA megadrought. The top lines show mean daily temperature in red and orange, and the lower bars show mean daily soil moisture content in light and dark blue for each microsite classification. Data models, statistical analyses, and visualization for this figure were done in R version 4.2.1 (see Supplement for full script and citation).

Vegetation was an important mediator of microclimate sampled during the SWNA megadrought (Fig. 2, Supplementary Fig. 1). Shrubs with an intact annual plant community of ground cover were an important microclimatic refuge in the winter 2017 with a significant lower mean annual temperature (Fig. 2, Non-linear mixed effects model, F-value (temp, microsite) = 4.682, *p* = 0.03, with estimated marginal means contrasts, *p* < 0.001). Ground cover vegetation mostly comprised of annual plants also influenced microclimate in non-canopied open microsites primarily in reducing soil moisture content (Fig. 2, Non-linear mixed effects model, F-value (moisture, treatment) = 158, *p* = 0.0001 with estimated marginal means contrasts, *p* < 0.001). Consequently, the most parsimonious ecological interpretation suggests that there are tradeoffs in the potential amelioration effects provided by resident plant species in the form of a native shrub foundation species and ground cover vegetation^36,37^. The canopy drives many processes locally in deserts^38,39^, and annual plants respond to soil moisture and precipitation patterns via drawdown and at times increased water use^40,41^. The synergistic or indirect benefits of facilitation of shrubs with an intact annual community^42,43^ provided the most significant returns on buffering through fine-scale patterning in plants suggest that complex direct and indirect community assembly processes^44^ influence climate mitigation capacities^45^. This is both novel and a critical research opportunity. Nonetheless, it is tempting to interpret differences between growing seasons and years instrumented here during the megadrought^46^. Mean annual temperature and precipitation differed in 2016 relative to 2017 for this region (2016 MAT 18.7 °C, MAP 215 mm; 2017 17.9 °C, 184 mm)^47^. However, limited snapshots in climatic series and inter-annual variability in drylands specifically^40,48^ suggest a more productive route to inform inference and decision making must include finer-scale estimates and more than one growing season. Variability is the norm not the anomaly—even within a single year between historically defined wet and dry seasons. Time-lag effects have also been detected in many grasslands globally^49,50^. We must manage for fine-scale heterogeneity through resident plant communities in dryland ecosystems because they provide crucial spatial heterogeneity (Fig. 3). Consistently lower or higher near-surface air temperatures were provided by foundation species to other resident species within this region. Gradual shifts in the microclimate within a season temporally coupled with the spatial heterogeneity in the vegetation tested here likely enables ‘tiny niches’ and shifting climate-envelope matching for both plants^21,51^ and animals—particularly ecotherms^52,53^. Temperature and soil moisture are but one set of many filters and capacities that we need to examine, and variability within a habitat is a resource and an asset^54^. Plants also provide other functions including carbon sequestration^9^ and resources directly for animals^55^. Further to the climate predictions here, shrubs can decouple some microclimatic measures but typically moved in concert with larger climatic trends and other vegetation present in the ecosystem. Buffering was detected but so were trade-offs induced by vegetation. The hypothesis that foundation species and other plants can enable fine-scale climate partitioning within drylands was thus supported.

Shrubs are the anchor of many mixed drylands. Canopy functions and stabilization by even relatively low stature but long-lived, ancient shrubs^29^ in many arid and semi-arid ecosystems can nonetheless provide fine-scale climate refuges^23^ and facilitate other species^32,56^. Similar to research in forests, canopy effects provide both direct and indirect buffering and mediation capacities from a rapidly changing climate both through heterogeneity and simple cover^57^. Unfortunately, all plant community composition measures in these ecosystems including grasslands globally can be negatively impacted by global change drivers^58^. Microclimatic approaches to modeling predictive responses to climate change at the ground level or plant and animal eye view in many systems to climate^51^ will be dramatically improved. The interaction between foundation plant species and climate will further provide a mitigation and management solution for stakeholders to consider in developing offset and conservation plans at novel and sensible scales. A generalized recent hierarchical conceptual workflow described by ‘protect-manage-restore’ for climate mitigation^59^, in that order, is thus strongly supported for dryland regions in California. First step, protect these relict native shrublands to provide microclimatic heterogeneity. This relatively passive investment in water resource infrastructure through vegetation is also one path forward to more effective water governance socially^60^. The patterns in vegetation-microclimate detected here need not always provide positive benefits on climate stressors in terms of direct amelioration but they did provide an important form of heterogeneity in climate at fine-scales that will reduce the risk or local extirpation and extinctions by providing species with options, or room to move, so as to access a variety of climatic regimes—particularly for species sensitive to temperature^61^. Planning and managing for microclimatic heterogeneity through both foundation plant species such as shrubs and ground-covering plants is thus a viable long-term solution in drylands facing megadroughts and other climate forcings associated with temperature and moisture.

## Methods

### Study site and species

Cuyama Valley is a mixed scrubland habitat located within the San Joaquin Desert in Central California, 34°56′53″ N 119°41′21″ W^26^. Water availability was already severely limited in this region in the last 60 years prior to the megadrought studied herein, and current work suggests further water deficits in precipitation, recharge, and groundwater with non-sustainable water use patterns predicted to continue under all human use and climate change scenarios^62^. The vegetation and climate spans arid to semi-arid temperatures and precipitation regimes consistent with generalized aridity patterns for most drylands globally^63,64^. There is thus a long history of groundwater measurement and management^65^. More recent studies report significant declines in groundwater availability^62,66^ and rapid declines in the elevation of the valley floor^67^. These patterns are associated with withdrawals from substantial irrigation^68^ and reduced inputs from precipitation during the megadrought^6^. US climate data from the region reports decreased precipitation in the years examined herein^69^. Additional climate data products queries confirmed these trends (i.e., Climate Research Unit, CRU version 4.05, high-resolution gridded datasets, and a single federal local weather station within valley). A total of 14 unique annual plant species, both native and exotic, were identified through vegetation censuses within the region during this study period^70^. The sole shrub species present regionally was *Ephedra californica* or Mormon Tea^71^. This native shrub is in the Gentales division and can reach heights of over 1 m^72^. It has a thready, diffuse canopy comprised of leaf-like needles similar to gymnosperms^73^. In spite of its limited canopy cover relative to other desert foundation shrub species such as Larrea species^74^, it functions as an ecological foundation species supporting other plants and animals^32,34,75^. Numerous small animals are present within the region including the US federally endangered species *Gambelia sila*^76^ that serves as an excellent flagship species within the state for many endangered desert species^77^. This vertebrate species and others have been shown to rely on *Ephedra californica*^54,78^.

### Experimental design

All accessible sites within the Cuyama Valley on public lands were surveyed for the presence of *Ephedra californica* the previous year (i.e. in 2015, over 700 km^2^ of drylands were censused for presence of this shrub species). Land classification maps were used to identify publicly designated lands. Every minimally pristine remnant patch or site of this native shrub species that was identified was instrumented in this study in 2016 and the winter of 2017. Pristine was parsimoniously defined as estimated effectively intact ecological function (i.e. shrubs present and with green tissue, and some native ground-cover vegetation present at the site), some evidence of resident biodiversity including native plant species and animal sign such as burrows, and these criteria were further coupled with evidence for relatively limited direct anthropogenic disturbances from agriculture or other activities within a 1 km^2^ area^79,80^ of a stand of shrubs. A site was defined as a relict stand of native shrubs with at least 30–50 intact and established adult individuals present within that constrained spatial extent. These relicts represent some of the extant distribution of this ancient shrub species, *Ephedra californica,* within the San Joaquin Desert and specifically the Cuyama Valley. A total of 180 adult shrubs were measured and assigned unique identifiers at the start of the growing season in January 2016. A total of 36 pairs of shrub-open microsites were then randomly selected from this comprehensive survey at independent sites at least 1 km^2^ in surface area that also met the additional criteria list above. Hobo 12-Bit Temperature Smart Sensors (https://www.onsetcomp.com/products/sensors/s-tmb-m0xx) with 10 cm soil moisture sensors (https://www.onsetcomp.com/products/sensors/s-smd-m005) were logged with sets of plug-and-play weather stations (https://www.onsetcomp.com/hobo-micro-station/). The reported and independently estimated error is + /− 3% for these specific sensors in field contexts^81^. This specific array deployment provides accurate near-surface air temperature and soil moisture contents relevant to ecological processes^82^. Measurements are at the scale of immediate and direct shrub canopy effects sampling up to 1 m^2^ of surface area ecologically for other plants and animals. A profile of temperature included up to 25 cm of near-surface air estimates, and soil moisture to a depth of 10 cm. These ranges are highly relevant to most annual plant species within the region. At each paired shrub-open microsite, all living aboveground plant material was removed by repeated clippings throughout the duration of microclimatic tracking. The removal of plant material complied with relevant national guidelines and legislation under the authority of the Bureau of Land Management, and no federally listed species were present^83^.

### Data and statistical models

All microclimatic data are publicly archived^84^. Data quality and control, exploratory data analyses with sensitivity analyses, and reported statistical models were done in R version 4.2.1^85^. Scripts are provided (Supplementary Text). Near-surface air temperatures and soil moisture did not demonstrate significant heteroscedasticity (*p* > 0.05 in Goodness-of-fit distributional testing), and missing values due to sporadic sensor failures were not imputed but coded as not available or NA in the R programming language. A total of 1,107,452 unique microclimatic measurements were recorded for the duration of the experiment (16 full months sampled). These data were summarized by hourly means for each sensor for each microsite instrumented (plotted in Fig. 2 as seasonal means per year for clarity and visualization of high-level patterns; however frequency histograms of the primary data are also provided in Supplementary Fig. 1). Season for this plot was defined using historical precedents of winter rains in the drylands of California, i.e. wet season wherein rainfall can potentially occur between November and April^47^. Models accounted for non-independence by nesting microsites within time (Supplementary Table 1). Site and microsite were modeled as random factors. Data were not modeled as unique pairs but treated as region-level estimate. Alternative data structures including all other measures of central tendency were examined and demonstrated robust trends to reported models including tests for season as a simple factor without year. Temperature and soil moisture data streams were examined individually using nonlinear-mixed effects models using the R package ‘nlme'^86^. The manipulative treatment of ground-cover vegetation was modeled as a fixed effect. Sensitivity analysis for data aggregation (hourly, monthly, seasonally, annually and spatially) with potential spatiotemporal autocorrelation dynamics were examined^87^. Model sensitivity was also examined by fitting mixed-effects, random models using the R package ‘lme4’^88^. The nonlinear-mixed models were robust and accurate. Post hoc contrasts were done using the R package ‘emmeans’^89^. This package computes contrasts and linear functions of the estimated marginal means derived from the nonlinear-mixed effect models. The climatograph shows the primary hourly data for the microclimatic data collected in this study.

## Supplementary Information

Supplementary Information.

## Data Availability

Data are freely available at Figshare. https://figshare.com/articles/dataset/Cuyama_micronet/11888199.
