# Supplementary material for: Foundation plant species provide resilience and microclimatic heterogeneity in drylands
Source: Sci Rep. 2022 Oct 26;12:18005. doi: 10.1038/s41598-022-22579-1 (PMC9606251; doi:10.1038/s41598-022-22579-1)
Supplement: Supplementary file 1 — Supplementary Information. [file 41598_2022_22579_MOESM1_ESM.pdf]

Supplement

Foundation plant species provide resilience and microclimatic heterogeneity in drylands

CJ Lortie [1*], Alessandro Filazzola [2], Mike Westphal [3], H. Scott Butterfield [4]

1. Department of Biology, York University, Toronto, ON, Canada. M3J1P3.
   ecodata@yorku.ca
   PH: 4167362100 FAX 4167365698 * **Corresponding author.**

2. Centre for Urban Environments, University of Toronto Mississauga, Mississauga, L5L 1C6, Ontario, Canada

3. Bureau of Land Management, Central Coast Field Office, 940 2nd Avenue, Marina, CA 93933, USA

4. The Nature Conservancy, 830 S Street, Sacramento, CA, 95811, USA.

Supplementary Table 1: Generalized linear mixed models (GLMMs) contrasting near-surface air temperatures temperature and soil moisture from microclimatic loggers by microsite location (shrub vs open), treatment (vegetation removed vs intact), and regional sites. The degrees freedom (numerator and denominator), F-value, *p*-value, and conditional R-squared value estimated from the fixed effects and random effects are reported. Microsite was nested within time and treated as a random effect.

Response	Predictor	DF	F-value	*p*-value	R^2
temperature	microsite	1, 190461	4.69	0.03	0.21
temperature	site	3, 190461	1112.1	<0.0001	0.21
temperature	treatment	1, 190461	21.9	<0.0001	0.21
soil moisture	microsite	1, 916975	1.00	0.32	0.19
soil moisture	site	5, 916975	2992.0	<0.0001	0.19
soil moisture	treatment	1, 916975	1584.9	<0.0001	0.19

Supplementary Figure 1. A contrast of the daily near-surface air temperatures and soil moisture content in Cuyama Valley, California in 2016 and the winter of 2017. Shrubs microsites are under the canopy of the resident native species Ephedra californica, and open are microsites without a woody canopy of a shrub. Clipped refers to the treatment of removing all aboveground non-woody vegetation both under shrubs and in the open. Two years were sampled at the peak of the SWNA megadrought. The frequency histograms show the distribution of the cumulative count of mean daily values by relative measures for each factor and microsite. Data models, statistical analyses, and visualization for this figure were done in R version 4.2.1.

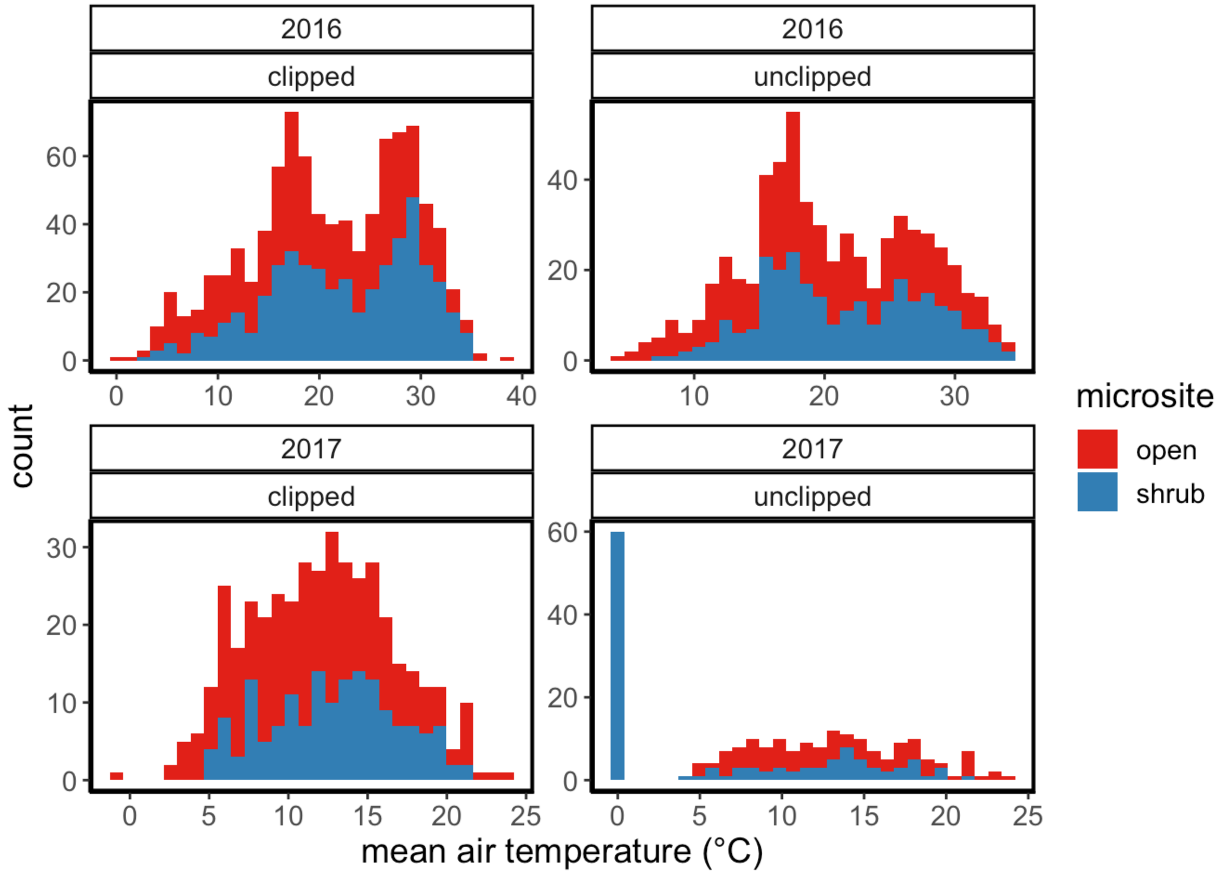

2016
clipped
2016
unclipped
2017
clipped
2017
unclipped
count
mean air temperature (°C)
microsite
open
shrub

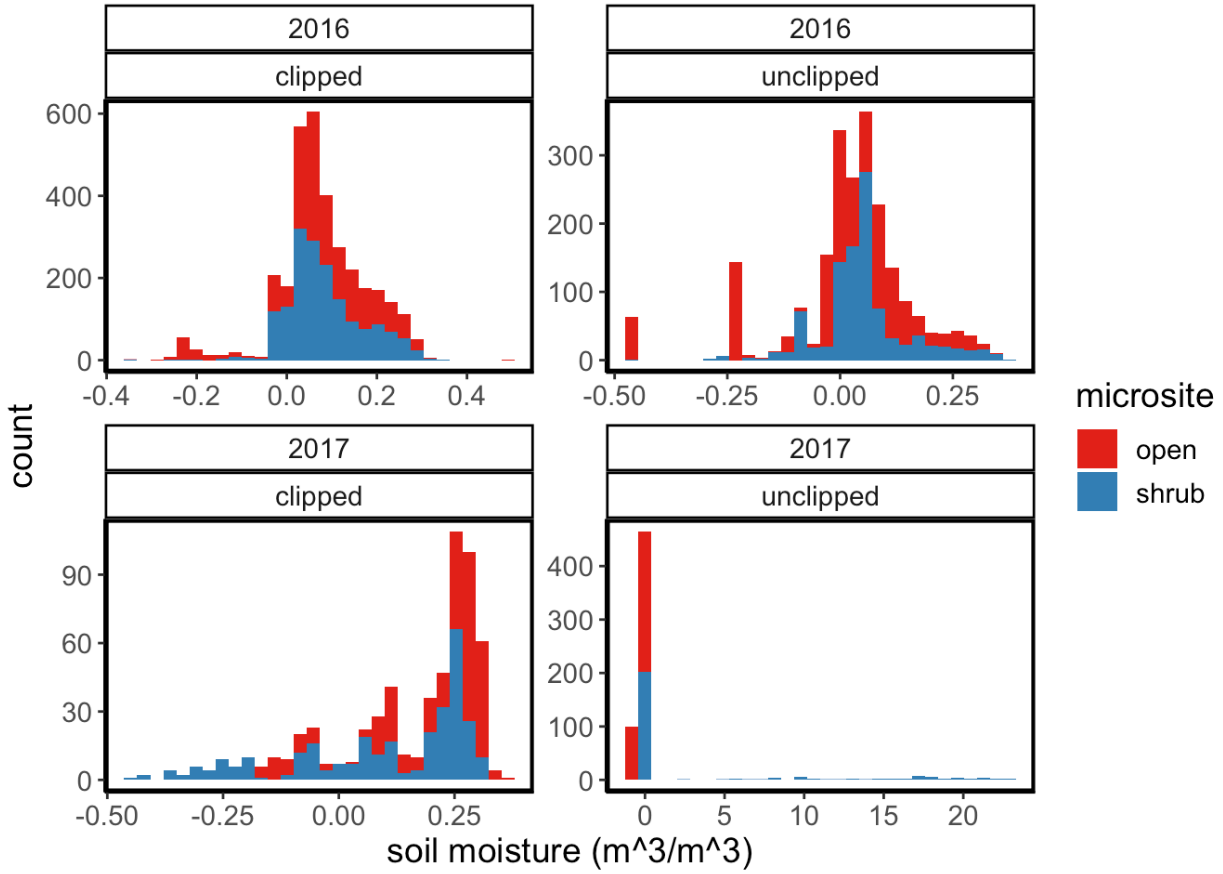

2016
clipped
2016
unclipped
2017
clipped
2017
unclipped
count
soil moisture (m^3/m^3)
microsite
open
shrub

Supplementary Text. The R open-source programming script for the data aggregation, primary data visualization, and main models. R Core Team (2022). R: A Language and Environment for Statistical Computing. Vienna, Austria, R foundation for Statistical Computing (https://www.R-project.org). Version 4.2.1 was used.

```
#### Data

```{r}

#primary data

library(tidyverse)

data <- read_rds("data/Cuyama_micronet.rds") %>%

  mutate(site = as.character(site), year = as.character(year)) %>%

  mutate(net = case_when(treatment == "clipped" & microsite == "shrub" ~ "shrub", treatment == "unclipped" & microsite == "shrub" ~ "shrub + vegetation", treatment == "clipped" & microsite == "open" ~ "open", treatment == "unclipped" & microsite == "open" ~ "vegetation"))


#derived data

data_interactions <- data %>%

  group_by(year, microsite, treatment, sensor_type) %>%

  summarize(m = mean(value), var = se(value), cv = abs(cv(value))) %>%

  mutate(sensor_type = case_when(sensor_type == "temp" ~ "air temperature", sensor_type == "soil moisture" ~ "soil moisture"))


#derived summary data

data_daily <- data %>%

  group_by(year, site, month, day, microsite, treatment, sensor_type) %>%

  summarize(m = mean(value), var = se(value), cv = abs(cv(value)))


#data lengths
```
```

````
dls <- data_daily %>%
  group_by(year, month) %>%
  summarise(n = n())

knitr::kable(dls)

days <- data_daily %>%
  group_by(year, month, day) %>%
  summarise(n = n())

length(days$day)

```

#### Viz
```{r}

#high-level two-factor plus model
ggplot(data_interactions, aes(microsite, m, color = treatment)) +
  geom_line(aes(group = treatment)) +
  geom_point(size = 3, shape = 18) +
  geom_errorbar(aes(ymin= m- var*1.95, ymax=m + var*1.95), width=.1) +
  facet_wrap(~year*sensor_type, scales = "free") +
  scale_color_brewer(palette = "Set1") +
  labs(y = "mean air temperature (°C) & moisture (m^3/m^3)", color = "") +
  theme_classic() +
  theme(text = element_text(size=14), panel.border = element_rect(colour = "black",
fill=NA, size=1.5))

#mean daily frequencies
````

~~~
data_daily %>%
  filter(sensor_type == 'soil moisture') %>%
  ggplot(., aes(m, fill = microsite)) +
  geom_histogram() +
  facet_wrap(~year + treatment, scales="free") +
  scale_fill_brewer(palette = "Set1") +
  labs(x = "soil moisture (m^3/m^3)") +
  theme_classic() +
  theme(text = element_text(size=14), panel.border = element_rect(colour = "black",
fill=NA, size=1.5))


data_daily %>%
  filter(sensor_type == 'temp') %>%
  ggplot(., aes(m, fill = microsite)) +
  geom_histogram() +
  facet_wrap(~year + treatment, scales="free") +
  scale_fill_brewer(palette = "Set1") +
  labs(x = "mean air temperature (°C)") +
  theme_classic() +
  theme(text = element_text(size=14), panel.border = element_rect(colour = "black",
fill=NA, size=1.5))

```


#### Models
```{r}
library(nlme)
library(emmeans)

#year as random
~~~

```
#temp

m1 <- lme(value ~ microsite + site + treatment, random = ~microsite*1|year, data =
subset(data, sensor_type == "temp"))

anova(m1)

coef(m1)


#contrast random model to fixed

m1.fixed <- gls(value ~ microsite + site + treatment, data = subset(data, sensor_type ==
"temp"))

anova(m1, m1.fixed)


#contrasts

m1.1 <- emmeans(m1, pairwise ~ microsite*site)

m1.1

#pairs(m1.1)


#soil moisture

m2 <- lme(value ~ microsite + site + treatment, random = ~microsite*1|year, data =
subset(data, sensor_type == "soil moisture"))

anova(m2)

coef(m2)


#contrast random model to fixed

m2.fixed <- gls(value ~ microsite + site + treatment, data = subset(data, sensor_type ==
"soil moisture"))

anova(m2, m2.fixed)


#contrasts

#contrasts

m2.1 <- emmeans(m2, pairwise ~ microsite*site)

m2.1
```

#pairs(m2.1)
```
```
